# Bridging the gap in cervical cancer screening for underserved communities: MCED and the promise of future technologies

**DOI:** 10.3389/fonc.2024.1407008

**Published:** 2024-07-29

**Authors:** Aya Hasan Alshammari, Hideshi Ishii, Takaaki Hirotsu, Hideyuki Hatakeyama, Masayo Morishita, Eric di Luccio

**Affiliations:** ^1^ Shonan Research and Development Center, Hirotsu Bio Science Inc., Tokyo, Japan; ^2^ Department of Medical Data Science, Center of Medical Innovation and Translational Research, Osaka University Graduate School of Medicine, Suita, Japan

**Keywords:** screening, MCED tests, disparities, early detection, underserved communities, cervical cancer

## Abstract

Cervical cancer screening is a critical public health measure, especially vital for underserved communities where disparities in access and outcomes are pronounced. Despite the life-saving potential of regular screening, numerous barriers—including geographical isolation, cultural and linguistic challenges, and socioeconomic factors—severely hinder accessibility for these populations. Multicancer early detection (MCED) tests emerge as a potentially effective intervention, offering a less invasive, more accessible approach that could transform how screenings are conducted. This paper explores the existing challenges in traditional cervical cancer screening methods, the potential of MCED tests to address these barriers, and the implications of these technologies for global health equity. Through a comprehensive review, we highlight the need for culturally sensitive, tailored interventions and the importance of effectively overcoming logistical and financial difficulties to implement MCED tests. Despite the promise shown by MCED tests, the paper acknowledges significant implementation challenges, including cost, logistical obstacles, and the need for cultural acceptance and validation studies. This study emphasizes the necessity for equitable MCED test implementation strategies, highlighting the potential of these innovative technologies to advance global health equity in cervical cancer prevention.

## 1 Introduction

Despite its potential to save lives, cervical cancer screening remains inaccessible to individuals in underserved communities (1). This disparity is especially concerning as cervical cancer, though highly preventable through screening (2), disproportionately burdens underserved populations. Cervical cancer screening, a cornerstone of preventive healthcare, enables the early detection of malignancies and potentially life-saving interventions (3). Nevertheless, the efficacy of cervical cancer screening hinges on addressing the complex and interconnected barriers faced by diverse communities. Such barriers could include limited clinic access, cultural beliefs, language constraints, or socioeconomic factors (4). This literature review focuses on underserved communities, such as those in remote regions, minority groups facing cultural barriers, or areas with limited medical infrastructure.

These disparities are especially pronounced in preventive health measures like cancer screening, often impeded by cultural beliefs, language barriers, and religious constraints (5, 6). Understanding and addressing these disparities is vital to improving public health (7, 8). We will first survey existing screening modalities to explore potential solutions, emphasizing their importance and limitations. Our investigation then moves to the disparities in screening rates among underserved communities and how these may hinder the primary goal of reducing the cancer burden in these groups. While acknowledging these challenges, we also examine the potential of multicancer early detection (MCED) tests, which aim to detect multiple cancers with a single, non-invasive test. With their non-invasive nature, MCED tests could potentially address some traditional barriers (9, 10). However, successful implementation in underserved settings will require careful research into their efficacy within specific populations alongside culturally tailored approaches.

## 2 Cervical cancer screening methods

### 2.1 Pap smear (cervical cytology)

While the Pap smear has a proven track record in detecting cervical dysplasia, its effectiveness relies on accurate interpretation, which can be subjective (11, 12). Regular screenings with Pap smears can proactively identify and manage these abnormalities before they develop into cervical cancer, underscoring the importance of routine tests in cancer prevention (2, 13, 14). Pap smear accessibility and cost-effectiveness facilitate broader screening (15, 16). On the downside, Pap smears can sometimes produce false-positive results. These inaccuracies can lead to unnecessary follow-up tests, resulting in anxiety and possibly financial challenges for patients (14, 17). There is also a concern that some precancerous lesions might go undetected (18). These limitations are particularly concerning in low-resource settings, where expertise in cytologic interpretation may be limited, hindering the effectiveness of Pap smears as a primary screening method (19, 20).

### 2.2 HPV testing

Persistent infections with high-risk HPV types can lead to cellular changes in the cervix that may progress to cancer (21). Thus, HPV vaccination can play a vital role in preventing cervical cancer (22). HPV testing is a more recent addition to cervical cancer screening. It excels in identifying high-risk HPV types that have been linked to cervical cancer (14, 23). As a testament to its efficacy, some protocols have begun using HPV testing as the primary screening method, streamlining the process (23–25). An added advantage is the potential for patients to collect their own samples. This self-sampling can enhance accessibility, especially in under-resourced areas (20, 23, 24). However, HPV testing is not without limitations. It often necessitates greater infrastructure requirements than Pap smears, which can be challenging in areas with limited resources (23, 26). While self-sampling has its merits, the availability and awareness of such kits can be inconsistent, potentially limiting their reach (27–29). Existing disparities in healthcare access can further affect the adoption and follow-up processes associated with HPV testing (30, 31).

### 2.3 Visual inspection with acetic acid

Visual inspection with acetic acid, commonly referred to as VIA, stands out as a screening technique due to its ability to yield immediate results (32). These immediate results facilitate real-time decision-making regarding any necessary treatment or further diagnostic tests, proving essential in many clinical settings (33). Financially, VIA is appealing as a low-cost method, offering an advantageous option for cervical cancer screening, especially in resource-constrained environments (32). Its simplicity and the absence of a requirement for any advanced technological equipment further enhance its appeal (32). However, VIA has its challenges. The accuracy of this method often depends on the proficiency and experience of the healthcare provider, introducing an element of subjectivity and potential variability in results (32).

### 2.4 Colposcopy

Colposcopy serves as an indispensable tool in the detailed examination of the cervix. The procedure provides a magnified and well-illuminated view of the cervix, enabling healthcare providers to precisely detect and inspect any abnormalities (34). If, during the process, any suspicious areas are identified, colposcopy offers the advantage of facilitating targeted biopsies, ensuring a focused examination (34). With skilled practitioners, colposcopy possesses a high level of accuracy in detecting cervical abnormalities. On the downside, the procedure is more invasive than some of its counterparts, which might induce discomfort or anxiety in certain patients (35, 36). Moreover, false negatives exist, implying that despite the enhanced view, some precancerous lesions or concerning areas might escape detection (35). Cost and equipment considerations also limit its widespread availability (37).

### 2.5 Cervical biopsy

The cervical biopsy procedure is the basis for a definitive diagnosis regarding cervical health (38, 39). It empowers medical professionals to make targeted treatment decisions based on the unambiguous presence or absence of precancerous or cancerous cells (38). The methodology behind a cervical biopsy is precise; the extracted tissue undergoes a thorough examination under a microscope, revealing detailed insights into cellular abnormalities (38). However, its invasive nature implies that it involves removing a tissue fragment from the cervix, which can also cause significant emotional strain for the patient. Additionally, there is an inherent risk of infection or bleeding post-procedure (40). Another concern revolves around the waiting period for the results. Unlike VIA, the outcomes from a cervical biopsy are not instantaneous, potentially leading to an unsettling period of anticipation for patients (40). Furthermore, depending on the location, there is a latent risk of missing the area with the most pronounced abnormality (40).

Despite the existence of these effective screening methods, significant disparities persist in who receives them. This uneven access to preventive care, particularly among underserved communities, is a critical public health concern that demands innovative solutions. The following section explores these disparities in detail, highlighting the geographical, cultural, linguistic, and social factors that create substantial barriers to cervical cancer screening.

## 3 The disparities in cervical cancer screening rates among underserved communities

This section underscores how access to life-saving cervical cancer screening varies substantially along lines of geography, culture, language, and religion. While disparities exist across nations, underserved communities within high-income countries also face barriers (41–43).

### 3.1 Geographical disparities

Geographical disparities drastically influence access to cervical cancer screening, leading to delays or complete absence of this vital preventive measure. This inequality significantly contributes to adverse health outcomes (44). Women in remote areas, low- and middle-income regions, and even specific locations within high-income countries experience substantial barriers due to distance from clinics, limited transportation, and inadequate healthcare facilities (45, 46). These disparities directly translate into higher cervical cancer incidence rates and poor survival outcomes in underserved areas (44).

The complexity of the issue is evident on both global and local scales. In China, geographical and socioeconomic inequities between regions fuel a high burden of cervical cancer (47). Even in the United States, despite targeted interventions like those in Maryland, significant geographical disparities in cancer mortality persist (48). This emphasizes how location, access to healthcare, and socioeconomic factors intertwine to influence individuals’ likelihood of receiving preventive screenings.

Certain populations face compounded challenges. Individuals with disabilities (49–51) and refugees (52) often encounter even greater difficulty accessing healthcare due to physical, linguistic, and systemic obstacles that are exacerbated by their geographical location. Addressing these complex issues requires a nuanced understanding of how different factors intersect to create barriers for specific groups.

Innovative approaches offer hope but necessitate careful implementation. HPV self-sampling holds promise for reaching underserved communities by reducing the need for in-person clinic visits (53). However, the sustained effectiveness of such interventions in improving outcomes must be assessed in the context of existing geographic disparities. Technologies like MCED, with their potential for less invasive sampling, could also help reduce geographical barriers; however, their efficacy in diverse real-world settings needs thorough investigation. While technology can be a powerful tool, it must be integrated with strategies that enhance healthcare infrastructure, address transportation issues, and provide tailored outreach to truly overcome geographic disparities in screening access.

In conclusion, geographical disparities play a major role in the preventable suffering caused by cervical cancer. Evidence underscores the urgent need for comprehensive strategies that address the root causes of these disparities (44, 45, 47, 48, 52, 53). These strategies must prioritize equity by improving healthcare infrastructure, tailoring education and awareness initiatives to specific regions, and leveraging technology to make screening more accessible for all.

### 3.2 Cultural beliefs and perceptions

Cultural beliefs and norms significantly influence how individuals perceive and approach healthcare decisions, particularly regarding sensitive topics like cervical cancer screening. Sociocultural factors, including religious beliefs, taboos around cancer, concerns about modesty, fear of familial judgment, and limitations on women’s decision-making autonomy, can all create substantial barriers to screening (54–56). These factors can lead to reluctance to seek care, even when symptoms are present.

This influence of culture on health behaviors is especially evident in low- and middle-income countries, where cultural values are deeply ingrained in how communities understand disease and interact with healthcare systems. In Uganda, for instance, cultural concerns about the HPV vaccine’s potential impact on fertility have hindered its acceptance, demonstrating how deeply held beliefs can supersede scientific evidence (57, 58).

Importantly, cultural barriers are not limited to low- and middle-income countries. Even within high-income nations like the United States, cultural and socioeconomic factors shape cervical cancer screening behaviors. Persistent misconceptions about the HPV vaccine’s safety, sometimes rooted in cultural beliefs, continue to impede preventive efforts (59–61). While innovative technologies like self-sampling or MCED might address concerns about privacy and invasiveness, overcoming deeply held misconceptions requires more than just technology.

Addressing the complexities of cultural barriers necessitates community-driven approaches. Utilizing community-based participatory research and developing culturally sensitive educational campaigns will be critical for increasing understanding and fostering trust within diverse communities. Achieving increased acceptance of cervical cancer screening requires acknowledging cultural differences and tailoring interventions accordingly.

### 3.3 Language barriers

Language barriers pose a significant obstacle to cervical cancer screening participation among culturally and linguistically diverse (CALD) populations. Challenges in accessing healthcare information and services due to language differences are a primary factor in the lower screening rates observed in these groups (62, 63). This underscores the broader challenges faced by CALD communities in accessing essential healthcare.

Research consistently demonstrates the impact of language barriers. In Hong Kong, limited language proficiency contributed to low screening rates among South Asian women, alongside broader informational gaps (62). Similarly, immigrant Muslim women face language-driven barriers in their decision-making about cervical cancer screening (64). This aligns with findings from other nations, where language is a recognized factor hindering screening access (65).

Healthcare providers are acutely aware of these challenges. General practitioners emphasize the need for interpreter services and culturally appropriate resources to better engage with CALD women during cancer screening programs (66). Studies from countries like the Netherlands further illustrate how language barriers lead to reduced screening participation and limit informed decision-making among Turkish and Moroccan women (67).

Language issues are recognized as health equity concerns even in countries like Canada, which have strong healthcare systems. Immigrants with limited fluency often encounter difficulties navigating the healthcare system, negatively impacting their access to preventive screenings (42, 68, 69).

To address these disparities, comprehensive solutions are needed. Healthcare systems must prioritize multilingual information, interpreter services, and culturally sensitive care. Technology, such as translation tools in MCED, could play a role but must be integrated with community outreach efforts that build trust and ensure information is tailored to the specific needs of CALD communities. Only by taking decisive action to overcome language barriers can we achieve equitable healthcare and improve cervical cancer screening outcomes for all.

### 3.4 Religious and social norms

Religious and social norms significantly influence cervical cancer screening uptake, particularly in culturally diverse settings. These norms shape women’s health behaviors and can result in screening delays or non-compliance. While innovative technologies like MCED offer the potential to reduce some barriers due to their less invasive nature, their successful implementation still requires a culturally sensitive approach.

Religious beliefs significantly influence attitudes toward healthcare interventions, including cervical cancer screening. In Zimbabwe, cultural and religious views, alongside insufficient knowledge and perceived stigma, hindered women’s participation in early screening (59). Similar challenges faced by immigrant Muslim women highlight the widespread impact of religious values on health-seeking behavior (64). However, integrating religious considerations into health messaging, as demonstrated by a faith-based intervention in Scotland, has the potential to mitigate these barriers and increase screening acceptance (70).

Social norms also play a powerful role. Women often underestimate the extent to which their peers engage in cervical cancer screening, suggesting that negative social perceptions can discourage individuals from seeking preventive care (71). Studies exploring norms related to cervical cancer screening, from Botswana to Singapore, further emphasize how gender roles, expectations, and broader social beliefs shape individual choices about screening (72, 73).

Targeted education interventions have shown promise in addressing the barriers posed by both religious and social norms. In Iran, theory-based education positively impacted knowledge and attitudes toward cervical cancer screening (74). Similarly, culturally tailored educational videos effectively improved understanding and screening intentions among Turkish- and Moroccan-Dutch women (75). Addressing cultural sensitivity and tailoring information is crucial, as seen in studies on breast and cervical cancer screening among immigrant populations in the United States (76).

In conclusion, religious and social norms significantly impact cervical cancer screening behaviors. Successful interventions must be designed with an understanding of the target population’s specific cultural, religious, and social context. These initiatives should focus on increasing knowledge, challenging harmful misconceptions, and leveraging positive social influences to encourage screening. By prioritizing community-driven approaches and tailoring healthcare solutions, we can make significant progress toward reducing cervical cancer disparities. Innovative technologies, such as MCED tests, offer the potential to address some of the traditional barriers faced by underserved communities. The following section will explore how MCED could substantially improve cervical cancer screening outcomes and propose strategies for overcoming the challenges in its implementation.

## 4 Proposing the use of MCED tests for cervical cancer screening in diverse communities

This proposal investigates how MCED tests have the potential to revolutionize cervical cancer screening outcomes, especially in underserved communities. These communities, including those in remote regions, recent immigrant populations, and areas with low socioeconomic status, face significant disparities due to limited clinic access, cultural barriers, and financial constraints (43, 65, 77–80). The disproportionate burden of cervical cancer in these communities underscores the urgent need for innovative solutions (81–84). Moreover, sample collection could potentially happen at home, further enhancing accessibility.

MCED tests, which analyze blood, urine, or other bodily fluids for a variety of cancer biomarkers, hold promise for overcoming traditional screening barriers. Their non-invasive sampling methods reduce the need for in-person clinic visits, addressing significant obstacles for those in remote areas where travel is difficult (85–87).

Cultural sensitivities, such as the stigma surrounding invasive procedures like Pap smears, can also be addressed by the less invasive nature of MCED. This offers a potentially more culturally acceptable alternative, allowing tailored interventions within diverse communities (88, 89). Successful initiatives like the Pitt County Breast Wellness Initiative-Education highlight the effectiveness of combining non-invasive methods with community-led outreach to increase screening rates (90).

While the specific application of MCED technology to cervical cancer is still in its early stages, its potential is promising. The non-invasive nature of MCED sampling methods offers the possibility to overcome numerous barriers underserved communities face. Simulation studies suggest MCED’s potential to reduce cancer mortality (91), and its effectiveness in detecting multiple cancers is established (92). However, dedicated clinical validation studies are needed to confirm its specific efficacy for cervical cancer detection. Nonetheless, its potential to address challenges like stigma, cultural barriers, and accessibility warrants focused research in underserved settings. Pilot programs within specific communities will be crucial to determine if MCED can effectively reduce cervical cancer screening disparities. Should MCED prove effective, its multicancer detection capability (91) could significantly improve health outcomes in populations with limited healthcare access.

This proposal recognizes the complexities of implementing MCED in under-resourced areas. These include cost, logistical difficulties, and ensuring culturally competent approaches. Prioritizing validation studies, alongside focused pilot programs exploring implementation feasibility, is vital. Multisector collaboration, guided by community input, will be essential to ensure that MCED benefits underserved communities and promotes global health equity in cancer care. The following sections will explore these implementation challenges and outline strategies for addressing them.

## 5 Challenges of implementing MCED tests in under-resourced areas

While MCED tests offer significant potential for transforming cervical cancer screening in under-resourced areas, their implementation faces substantial challenges (93–96). These include cost, cultural acceptance, logistical complexities, and ethical considerations. In underserved communities, these challenges are amplified by limited resources, infrastructure constraints, and unique cultural and socioeconomic factors (94). Ensuring the effectiveness of MCED across diverse populations is crucial, with research highlighting the need for cancer screening approaches to be sensitive to cultural and genetic variations (97). Additionally, MCED tests raise ethical concerns, particularly in low-resource settings, regarding how to communicate results in a way that reduces anxiety and, importantly, how to ensure access to appropriate follow-up care (10).

Addressing cost is essential for MCED to be widely accessible in underserved communities. Government subsidies and volume guarantees are vital policy solutions. Alongside these, community-led initiatives could focus on awareness campaigns to address transportation barriers, culturally tailored outreach, and partnerships with local organizations for on-site screening or subsidy distribution (98). As unforeseen obstacles may arise, successful implementation will require flexible, iterative approaches developed in collaboration with communities.

Logistical difficulties, such as equipment distribution and maintenance, pose additional barriers to MCED adoption, especially in remote or under-resourced clinics. Emerging technologies, like blockchain and smart contracts, could potentially streamline supply chains and enhance trust (99). However, for these solutions to be effective, they must be adapted to the realities of clinics in underserved areas, ensuring compatibility and ease of use within existing infrastructure.

**Figure 1:**
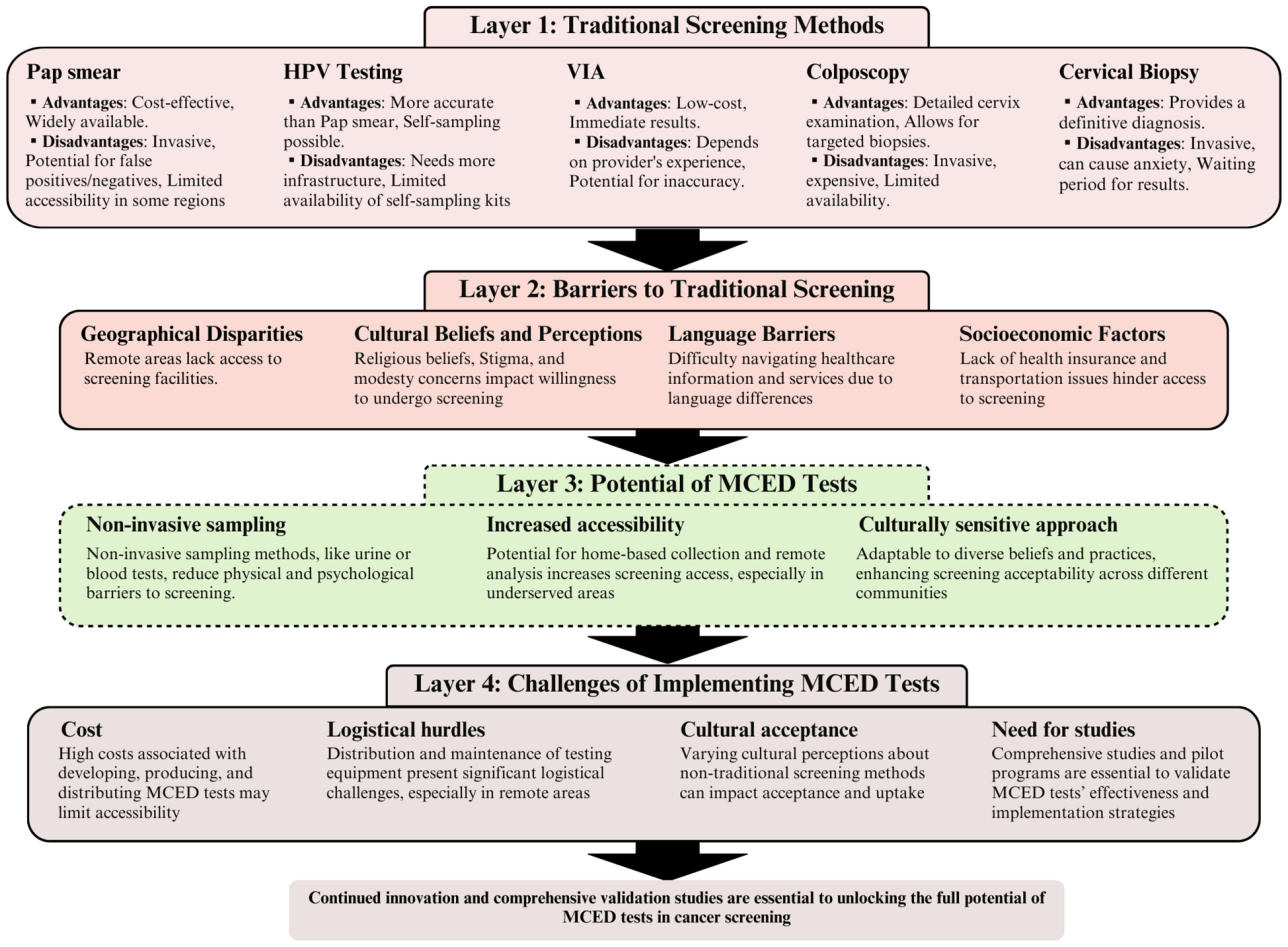
The evolution of cervical cancer screening: from traditional methods to multicancer early detection (MCED) innovations. This figure illustrates a four-layered analysis of cervical cancer screening strategies, beginning with traditional screening methods (layer 1), progressing through the barriers to these traditional screenings (layer 2), the potential benefits of MCED tests (layer 3), and concluding with the challenges associated with implementing MCED tests (layer 4). Each layer employs brief descriptions to represent key concepts, methods, barriers, and benefits, connected through a series of arrows to demonstrate the flow and relationships between layers. The figure concludes with a call to action, pointing toward the need for further research and pilot programs in the journey toward the effective implementation of MCED tests in cervical cancer screening.

Addressing these multifaceted challenges requires a genuinely collaborative approach. Technological innovation, policy changes that specifically target the unique needs of underserved communities, and initiatives driven by community input and leadership must work in concert to ensure that the life-saving benefits of MCED reach those most in need. The following sections will propose strategies to overcome these obstacles, drawing inspiration from successful health initiatives in diverse contexts (**Figure 1**).

## 6 Strategies for overcoming the challenges of implementing MCED tests in underserved communities

### 6.1 Addressing cultural barriers through community partnerships

In underserved communities, addressing cultural barriers is crucial for successfully implementing MCED. Building trust through community-led education and addressing misconceptions are key components of this approach. Technology offers innovative ways to amplify these efforts.

Collaborating with organizations like the Partnership for Native American Cancer Prevention (NACP) highlights the importance of engaging community leaders. Their expertise in developing culturally sensitive messaging and outreach fosters trust and promotes participation (100). Additionally, targeted initiatives, such as student-led educational programs modeled on those used for skin cancer awareness, can increase overall health literacy and empower individuals from diverse backgrounds to make informed decisions about their health (101).

Technology may significantly expand the reach of these educational efforts. Adapting platforms like Shezlong, an online mental health platform offering virtual therapy services, can potentially deliver tailored MCED information to underserved populations and could address specific concerns and potential misconceptions (102). However, for these tools to be effective, they must be designed with an understanding of the cultural context, technological literacy, and the practical limitations faced by these communities.

### 6.2 Government subsidies and financial support

Government subsidies integrated into national health schemes are essential for making MCED tests accessible to underserved communities. Even when diagnostics are nominally covered, out-of-pocket costs remain a substantial barrier for low-income individuals, as evidenced by analyses across several Asian countries (103). Successful subsidy programs must address the cost of MCED tests and associated logistical expenses, such as transportation and potential follow-up care, that can deter participation.

Streamlining reimbursement systems within subsidized schemes is crucial to avoid overburdening clinics serving underserved populations. Inefficient processes create additional barriers for healthcare providers (104). The long-term cost-effectiveness of preventative care, including cancer screening, has been well-documented (105), strengthening the argument for upfront investments in MCED accessibility.

### 6.3 Partnerships with non-governmental organizations and international organizations

Strategic partnerships with non-governmental organizations (NGOs) and international organizations with expertise in healthcare delivery, technology deployment, and community engagement are essential for the successful and equitable implementation of MCED in underserved settings. These partnerships offer resources and insights vital to addressing complex logistical, technological, and trust-related barriers.

NGOs specializing in healthcare provision in remote or low-resource areas possess significant knowledge of how to overcome access challenges. Their experience in tailoring technology deployment to the specific needs of underserved populations, as emphasized in child health initiatives (106), will be crucial for MCED. The design of MCED interfaces, result-reporting systems, and any associated technologies must be done in close collaboration with these NGOs to ensure compatibility and functionality within the constraints of rural or underfunded clinics.

Building community trust is essential for adopting novel screening technologies like MCED. Collaborations with NGOs that have strong community connections and a history of addressing health misconceptions, such as those combating cancer stigma in Pakistan (107), will be essential. These NGOs can lead targeted awareness campaigns and facilitate open dialogue between communities and healthcare providers. To ensure ethical deployment and rigorous evaluation of MCED in diverse contexts, partnering with NGOs specializing in community-engaged research is vital (10). Their expertise will ensure diverse patient perspectives are included throughout the process, from early-stage technology adaptations to long-term impact assessments.

### 6.4 Leveraging technology and telemedicine

The utilization of technology and telemedicine presents a promising solution to overcome geographical barriers that limit MCED access in underserved areas. Telemedicine, the practice of providing healthcare services remotely via telecommunications technology, may substantially alter how MCED results are delivered and how patients are monitored.

Research demonstrates the feasibility of remotely transmitting MCED sample results to centralized labs equipped with specialized staff (108). This addresses a critical challenge in areas where local clinics may lack the expertise to interpret complex test results. Furthermore, telemedicine is essential for continuous patient monitoring after MCED screening (109), ensuring timely follow-up care—a crucial component of effective cancer screening, especially in communities where traveling long distances for appointments is burdensome.

To maximize the reach of telemedicine in MCED implementation, user-friendly interfaces are critical, especially in populations with varying levels of technological literacy. Collaborating with community organizations can ensure these interfaces are designed with the specific needs of underserved populations in mind. Encouragingly, there is growing acceptance of telemedicine among healthcare providers, which is vital for successful MCED integration (110). However, alongside technological innovation, it is essential to proactively address concerns around data privacy and establish sustainable reimbursement models within healthcare systems to ensure equitable, long-term access.

## 7 Conclusion

This review underscores the urgent need to transform cervical cancer screening approaches, particularly within underserved communities. MCED tests hold the potential to revolutionize screening access due to their ability to overcome many traditional logistical and cultural barriers. However, successful implementation requires concerted efforts to address financial constraints, optimize deployment strategies, and ensure cultural sensitivity.

Future research must prioritize the rigorous clinical validation of MCED technologies, solidifying their efficacy in early cancer detection (111, 112). Simultaneously, investigations into cost-effective and contextually adaptable implementation strategies for low-resource settings are imperative (113, 114). To address potential reluctance and misinformation, community engagement in developing culturally appropriate outreach programs is essential (115).

Reducing cervical cancer’s global burden, with a focus on marginalized populations, demands an integrated approach. Technological innovation alone is insufficient. Concurrent policy changes promoting equitable distribution and access to MCED are essential. Research and policy efforts must collaborate to create an environment where all individuals, regardless of location or socioeconomic circumstances, have access to life-saving cancer screening services.

## Author contributions

AA: Writing – review & editing, Writing – original draft, Visualization, Methodology, Investigation, Data curation, Conceptualization. HI: Writing – review & editing. TH: Writing – review & editing, Validation, Supervision. HH: Writing – review & editing. MM: Writing – review & editing. EL: Writing – review & editing, Validation, Supervision, Project administration.
